# Eukaryotic translation elongation factor 1 delta alleviates DNA damage during bovine alphaherpesvirus 1 infection

**DOI:** 10.4142/jvs.25212

**Published:** 2026-07-08

**Authors:** Xiuyan Ding, Xiaozhen Ma, Bin Hou, Liqian Zhu

**Affiliations:** Key Laboratory of Microbial Diversity Research and Application of Hebei Province, College of Life Sciences, Hebei University, Baoding 071002, China.

**Keywords:** DNA damage response, γH2AX, P53-binding protein 1, bovine alphaherpesvirus 1, cells, cultured

## Abstract

**Importance:**

Bovine alphaherpesvirus 1 (BoAHV-1) is one of the most important viruses that infects cattle and causes significant economic losses. Understanding the critical mechanisms underlying viral pathogenesis is essential for developing novel antiviral strategies.

**Objective:**

To determine if eukaryotic translation elongation factor-1 δ (EEF1D) is involved in DNA damage repair in the presence or absence of BoAHV-1 infection by regulating the DNA damage response (DDR) signaling of P53-binding protein 1 (53BP1) and γH2AX.

**Methods:**

Bovine kidney (MDBK) cells were transfected with EEF1D-specific small interfering RNAs (siRNAs) or a scrambled control siRNA, followed by either a mock infection or virus infection. The effects of EEF1D knockdown on the γH2AX protein levels, γH2AX foci formation, and 53BP1 protein expression and foci formation were evaluated by Western blotting and immunofluorescence assays.

**Results:**

siRNA-mediated EEF1D depletion increased the γH2AX protein levels irrespective of a viral infection and enhanced γH2AX foci formation in BoAHV-1–infected cells. In mock-infected cells, EEF1D knockdown increased the steady-state 53BP1 protein levels and 53BP1 foci formation. In contrast, during viral productive infection, EEF1D knockdown reduced the 53BP1 protein levels despite concurrently suppressing viral protein expression.

**Conclusions and Relevance:**

EEF1D appears to alleviate BoAHV-1-induced DNA damage, potentially by modulating 53BP1-related DDR signaling. Hence, EEF1D may be a host factor linking viral replication and DDR regulation and may contribute to BoAHV-1 pathogenesis.

## INTRODUCTION

Bovine alphaherpesvirus 1 (BoAHV-1) is an enveloped DNA virus belonging to the genus *Varicellovirus* in the subfamily *Alphaherpesvirinae* under the family *Herpesviridae* [1,2]. It is one of the most important viral pathogens affecting cattle farming [1,3,4]. Viral replication may generally induce mucosal lesions in the upper respiratory tract and suppress the host’s immune responses [5,6,7], leading to secondary infections by other pathogens. This can result in a severe disease known as the bovine respiratory disease complex [8]. BoAHV-1 infection accounts for 25%–60% of abortions that occur in cows [8]. Therefore, it causes substantial economic losses in the global cattle industry [2,9].

The eukaryotic translation elongation factor-1 (EEF1) protein complexes recruit aminoacylated transfer (t)RNA molecules to ribosomes in a GTP-dependent manner during protein synthesis [10]. The EEF1 complexes contain two subunits, EEF1A and EEF1B. EEF1δ (EEF1D) is a component of the EEF1B complex [11]. In addition to mediating protein translation, EEF1D has broad effects on various cellular processes, including the recognition of damaged proteins, activation of the proteasome degradation system, and regulation of cellular apoptosis [12]. Importantly, accumulating studies suggest that EEF1D proteins are often overexpressed and involved in several types of cancer, such as glioma, ovarian cancer, and oral squamous cell carcinoma [13]. Thus, EEF1D aberrant expression is pathogenic and potentially associated with a range of diseases. A previous study reported that EEF1D may associate with the BoAHV-1 viral protein ICP8, a key component of the viral replication compartment, suggesting a potential mechanism by which EEF1D facilitates viral infection processes [14].

Over-accumulated DNA damage, such as double-strand breaks (DSBs), can lead to genomic alterations that are responsible for many diseases [15]. BoAHV-1 infection induces DNA damage and affects multiple DNA damage response (DDR)-related molecules [16,17,18]. For example, it increased the formation of γH2AX foci but abolished the formation of P53-binding protein 1 (53BP1) foci [18]. Nevertheless, the role of EEF1D in virus-induced DNA damage and DDR signaling is unclear. Interestingly, Xu et al. [19] reported that the knockdown of EEF1D expression exacerbates by cis-dichlorodiammine platinum (DDP)-induced DNA damage in ovarian cancer cells, suggesting that EEF1D is potentially involved in DDP-induced DDR. The detailed mechanisms of how EEF1D regulates DDR remain to be determined. This study hypothesized that EEF1D may promote DNA damage repair and examined its role in BoAHV-1 infection-induced DNA damage.

## METHODS

### Virus and cells

Bovine kidney MDBK cells were purchased from the Chinese Model Culture Preservation Center in Shanghai, China. The cells were routinely passaged and maintained in Dulbecco’s Modified Eagle Medium supplemented with 10% fetal bovine serum. BoAHV-1 strain NJ-16-1, isolated from commercial bovine semen [20], was propagated in MDBK cells. Aliquots of the virus stocks were stored at −80°C for subsequent studies.

### Antibodies

EEF1D rabbit polyclonal antibody (pAb) (cat# A2509), Phospho-Histone H2AX(S139) (γH2AX) rabbit pAb (cat# AP0099), Histone H2AX rabbit pAb (cat# A11463), GAPDH rabbit pAb (cat# AC027), and β-tubulin rabbit pAb (cat# AC015) were supplied by Abclonal Technology (USA). The 53BP1 antibody (cat# NB100-304) was provided by Novus Biologicals (USA). Goat anti-BoAHV-1 serum (cat# PAB-IBR) was supplied by VMRD (USA). Horseradish peroxidase-labeled goat anti-rabbit IgG (cat# BF03008) was provided by Biodragon (China).

### EEF1D-specific small interfering RNAs (siRNAs)

siRNAs targeting EEF1D, specifically siEEF1D-1: GCAUGAGAAGAUCUGGUUUTT and AAACCAGAUCUUCUCAUGCTT, and siEEF1D-2: GCGUCGACAUUGCUGCUUUTT and AAAGCAGCAAUGUCGACGCTT, along with the scrambled siRNA, were provided by Genepharma (China) [14]. Transfection of these siRNAs was carried out using the siRNA-Mate transfection reagent from Genepharma, according to the manufacturer’s instructions.

### Western blot

The cell lysates were prepared using radioimmunoprecipitation assay lysis buffer, composed of 1× phosphate buffered saline (PBS), 1% NP-40, 0.5% sodium deoxycholate, and 0.1% sodium dodecyl sulfate (SDS), and supplemented with a protease inhibitor cocktail. These lysates were boiled in Laemmli sample buffer for 10 min, then separated by SDS-polyacrylamide gel electrophoresis (using 8% or 10% gels), and transferred to polyvinylidene fluoride membranes. Immunoreactive bands were visualized using the Clarity Western ECL Substrate from NCM Biotech (cat# P10300).

The band intensities were analyzed quantitatively using the Image J program (National Institutes of Health, USA). The statistical significance was determined using a Student’s *t*-test with GraphPad Prism software (version 9.0). The *p* values < 0.05 (denoted as ^*^p < 0.05) were considered significant.

### Immunofluorescence assay (IFA)

MDBK cells in 24-well plates containing coverslip were fixed with a 4% solution of paraformaldehyde in PBS for 10 min at room temperature, permeabilized with 0.25% Triton X-100 in PBS for 5 min at room temperature, and blocked with 1% bovine serum albumin (BSA) in PBST (PBS containing 0.1% Tween 20) for 1 h. Subsequently, the cells were incubated with the specified primary antibodies in 1% BSA in PBST overnight at 4°C. After three washes, the cells were incubated with secondary antibodies conjugated to different fluorescent dyes for 1 h in the dark at room temperature. Another three washes were performed, followed by DAPI (4′,6-diamidino-2-phenylindole) staining to visualize the nuclei. The coverslips were then mounted onto slides with an antifade mounting medium (cat# 50-247-04; Electron Microscopy Sciences, USA). The images were obtained using a confocal microscope (Zeiss, Germany).

## RESULTS

### EEF1D is potentially involved in the DDR

**Fig. 1:**
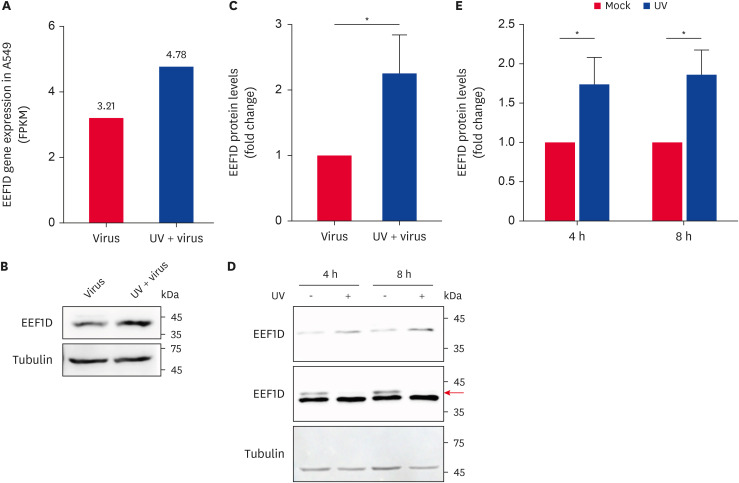
EEF1D expression in UV-treated and BoAHV-1–infected A549 cells. (A) A549 cells in 60 mm dishes were either mock-treated or treated with UV light for 4 min, and subsequently infected with BoAHV-1 (MOI = 1) for 4 h. The cells were then collected for RNA-seq analysis performed by BGI Genomics Co., Ltd., with the gene expression levels analyzed using the FPKM method. (B) A549 cells in 60 mm dishes were either mock-treated or treated with UV light for 4 min, and subsequently infected with BoAHV-1 (MOI = 1) for 24 h. The cells were collected for Western blot analysis to detect EEF1D using an EEF1D-specific antibody (catalog No. A2509, 1:1,000; Abclonal Technology). (D) A549 cells in 60 mm dishes were either mock-treated or treated with UV light for 4 min. They were incubated for 4 and 8 h, respectively. The cell lysates were prepared and subjected to Western blot analysis to detect EEF1D using an EEF1D-specific antibody (catalog No. A2509, 1:1,000; Abclonal Technology). The red arrow indicates a band with molecular weights higher than the expected EEF1D, which was recognized by the EEF1D-specific antibody. (C, E) The band intensities of EEF1D in the Western blot were quantified using the free software ImageJ. The intensity of each band was first normalized to that of the respective loading control Tubulin and then normalized to that of the mock-treated control, which was arbitrarily set to 1. The data shown are representative of three independent experiments, and the error bars represent the standard deviations of three independent experiments. Significance was assessed using a Student’s *t*-test. EEF1D, eukaryotic translation elongation factor-1 δ; UV, ultraviolet; BoAHV-1, bovine alphaherpesvirus 1; MOI, multiplicity of infection; FPKM, fragments per kilobase of transcript per million fragments mapped. ^*^p  <  0.05.

Ultraviolet (UV) irradiation-induced DDR in A549 cells was reported to differentially affect the expression of BoAHV-1 genes, including the infected cell protein 0 (bICP0), bICP22, and viral glycoprotein C (gC) and gD [21]. In response to UV-induced DDR, the host cells mobilize a series of molecules to modulate viral replication. This process provides an efficient method for identifying the host factors linking viral replication and DDR. Accordingly, virus-infected A549 cells, with or without UV irradiation, were subjected to RNA sequencing. As a result, several host proteins were differentially affected by the UV treatment in the virus-infected A549 cells (data not shown). Among them, the transcript levels of the EEF1D gene increased moderately after UV irradiation (Fig. 1A), which was confirmed by Western blot assay (Fig. 1B). Quantitative analysis showed that the EEF1D protein levels increased approximately 2.3-fold compared to the mock-treated, virus-infected control (Fig. 1C). Further studies suggested that UV irradiation alone (in the absence of viral infection) also influences EEF1D expression (Fig. 1D, upper panel). Specifically, the EEF1D protein levels increased to approximately 1.74- and 1.86-fold at 4 and 8 h post UV-irradiation, respectively (Fig. 1E). A faint band (denoted by an arrow) with a higher molecular weight was clearly observed after over exposure (Fig. 1D, middle panel), and its protein levels decreased dramatically in response to UV irradiation. This band may represent an EEF1D isoform because 4 EEF1D isoforms have been reported [13], which remains to be clarified. In summary, these studies show that UV irradiation increases EEF1D protein expression in cell cultures irrespective of BoAHV-1 productive infection.

EEF1D warrants further investigation because it is involved in RNA transcriptional and translational processes and regulates the sensitivity of ovarian cancer cells to cisplatin treatment [22]. Cisplatin can induce cell apoptosis by causing DNA damage [23]. Thus, it may be involved in DDR and in regulating viral infections. A previous study reported that EEF1D is indeed involved in BoAHV-1 productive infection, potentially by regulating the viral replication compartment in MDBK cells [14]. Since MDBK cells support viral replication more efficiently than A549 cells, and they are used widely for studying the virus replication mechanism, MDBK cells were used for further studies to determine if EEF1D is involved in the virus-infection-induced-DDR in the absence of UV-treatment.

### EEF1D is potentially involved in DNA damage

**Fig. 2:**
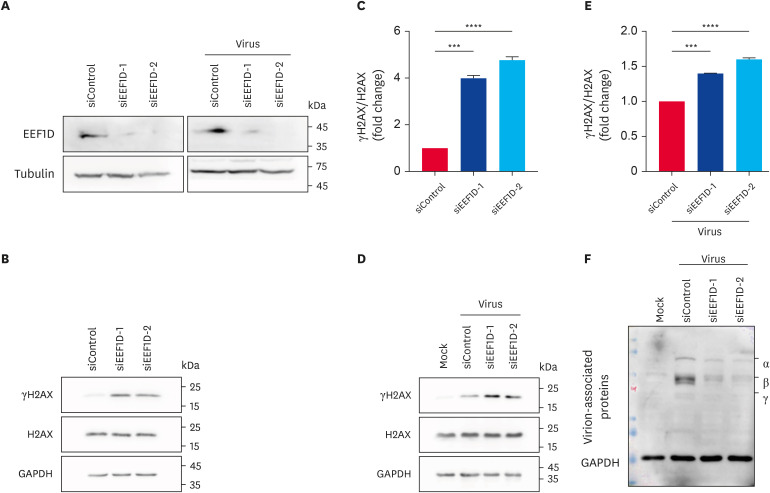
Detection of the γH2AX protein levels in response to EEF1D depletion. MDBK cells in 6-well plates were transfected with scrambled siRNA (150 pmol), siEEF1D-1 (150 pmol), or siEEF1D-2 (150 pmol). At 36 h post-transfection, the cells were either collected or infected with BoAHV-1 at an MOI of 1 for 24 h. The cells were collected and subjected to Western blot analysis to detect the protein levels of EEF1D (A), γH2AX, and H2AX (B, D), as well as virion-associated proteins (F). The antibodies used were against EEF1D-specific antibody (catalog No. A2509, 1:1,000; Abclonal Technology) (A), γH2AX (catalog No. AP0099, 1:1,000; Abclonal Technology) (B, D), H2AX (catalog No. A11463, 1:1,000; Abclonal Technology) (B, D), and goat anti-BoAHV-1 serum (catalog No. PAB-IBR, 1:5,000; VMRD) (F), respectively. (C, E) Band intensities in the Western blot assay were analyzed using the free software Image J. The intensity of each γH2AX band was first normalized to that of H2AX, and then normalized to that of the control transfected with scrambled siRNA, which was arbitrarily set to 1. The data are representative of three independent experiments, with error bars indicating the standard deviations of three independent experiments. The significance was assessed using a Student’s *t*-test. EEF1D, eukaryotic translation elongation factor-1 δ; si-, small interfering; BoAHV-1, bovine alphaherpesvirus 1; MOI, multiplicity of infection; GAPDH, glyceraldehyde 3-phosphate dehydrogenase. ^***^p <  0.001, ^****^p <  0.0001.

Previous studies have established that the histone variant H2AX is recruited to the sites of DSBs, where it is then phosphorylated at Ser139 residue (referred to as γH2AX) by DNA damage-activated kinases, such as ataxia-telangiectasia mutated (ATM), ATM- and Rad3-related (ATR), and DNA-dependent protein kinase (DNA-PKcs) [24,25,26]. γH2AX serves as a docking site for the recruitment of other DDR proteins, such as 53BP1, which are crucial for DNA damage repair [24]. Thus, immunostaining for γH2AX accumulated at DSBs appears as foci, which are generally considered the hallmarks of DNA damage [27,28]. Here, EEF1D-specific siRNAs, denoted by siEEF1D-1 and siEEF1D-2, respectively, could significantly knock down EEF1D protein expression in a context either with or without virus infection (Fig. 2A), as described elsewhere [14]. The transfection of these two individual siRNAs increased the γH2AX protein levels, but not that of H2AX, irrespective of virus infection (Fig. 2B and D). Quantitative analysis showed that the γH2AX protein levels increased approximately 3.98- and 4.76-fold in mock-infected cells, and approximately 1.40- and 1.60-fold in virus-infected cells transfected with siEEF1D-1 and siEEF1D-2, respectively (Fig. 2C and E). Although a BoAHV-1 productive infection leads to increased γH2AX levels [18], elevation of the γH2AX protein levels in response to EEF1D knockdown cannot be attributed to viral replication. This is because viral protein expression was significantly reduced by these two siRNAs (Fig. 2F). At least three virion-associated proteins, denoted by α, β, and γ, were reduced as probed by the antibody against virion-associated proteins (Fig. 2F). Specifically, the commercially available goat anti-BoAHV-1 serum used in this study was generated by immunizing goats with purified BoAHV-1 viral particles. As described elsewhere [14], this antibody detected several bands indicative of different viral proteins by Western blot. Although the specific viral protein corresponding to each band could not be identified, they can serve as indicators of virion-associated proteins.

**Fig. 3:**
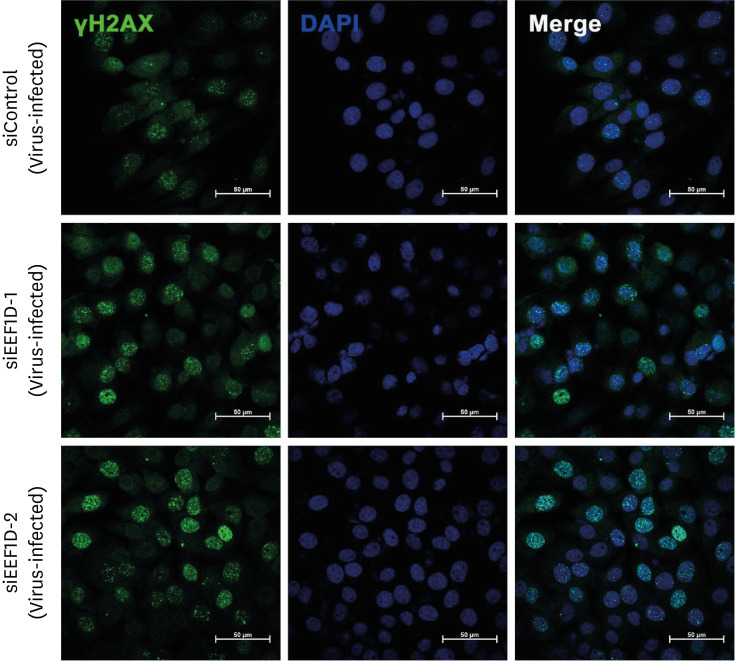
Effects of EEF1D depletion on γH2AX foci formation in virus-infected MDBK cells. MDBK cells were transfected with either scrambled siRNA (40 pmol) or siEEF1D (40 pmol), respectively. At 36 h post-transfection, the cells were infected with BoAHV-1 at an MOI of 1. Subsequently, γH2AX (green) foci were detected by immunostaining using a γH2AX-specific antibody (catalog No. AP0099, 1:800; Abclonal Technology). Nuclei were counterstained with DAPI (blue). Immunostaining was visualized, and the images were captured using confocal microscopy (Zeiss). Scale bars = 50 μm. The displayed fields are representative of three independent experiments. EEF1D, eukaryotic translation elongation factor-1 δ; si-, small interfering; BoAHV-1, bovine alphaherpesvirus 1; MOI, multiplicity of infection; DAPI, 4′,6-diamidino-2-phenylindole.

The IFA showed that the signal intensity of γH2AX foci, induced by BoAHV-1 infection, was significantly enhanced because of the knockdown of EEF1D protein expression by siEEF1D-1 and siEEF1D-2 (Fig. 3). These findings suggest that the knockdown of EEF1D expression enhances the DDR signaling of γH2AX, as evidenced by the increased protein levels of γH2AX and the formation of γH2AX foci, indicative of exacerbated DNA damage.

### Depletion of EEF1D expression enhances 53BP1 protein expression, an effect that is overridden by a BoAHV-1 infection

**Fig. 4:**
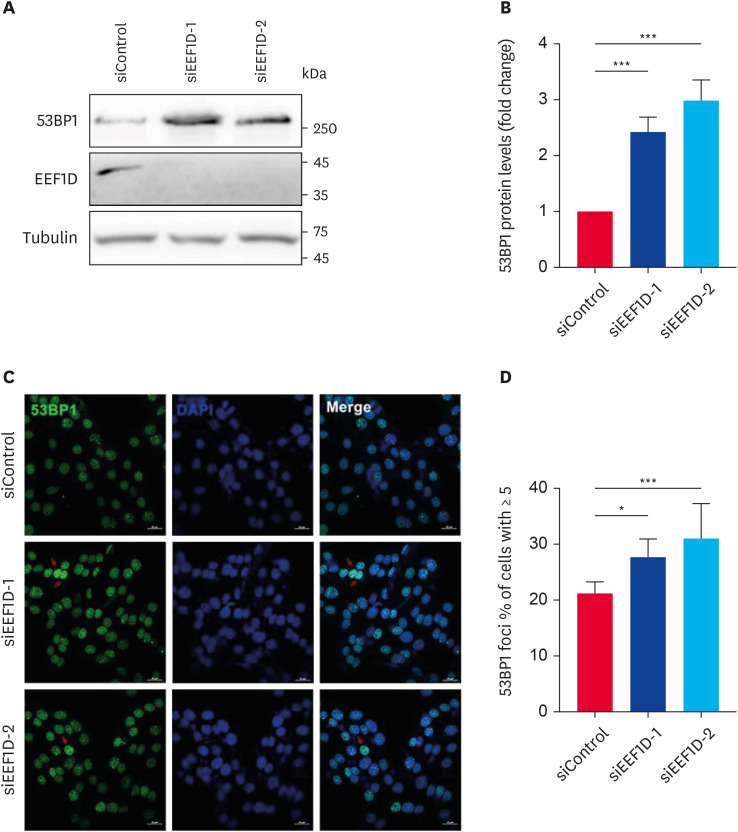
Effects of EEF1D depletion on 53BP1 protein expression and 53BP1 foci formation in mock-infected cells. (A) MDBK cells in 6-well plates were transfected with scrambled siRNA, siEEF1D-1, and siEEF1D-2, at a dose of 150 pmol, respectively. At 48 h post-transfection, the cells were collected for Western blot analysis using antibodies against 53BP1 (catalog No. NB100-304, 1:3,000; Novus Biologicals) and EEF1D (catalog No. A2509; 1:1,000; Abclonal Technology), respectively. (B) Band intensities were quantified using the free software Image J. The intensity of the 53BP1 band was first normalized to that of the respective loading control β-tubulin, and then normalized to that of the control transfected with scrambled siRNA, which was arbitrarily set to 1. (C) MDBK cells in 24-well plates containing coverslips were transfected with either scrambled siRNA (40 pmol) or EEF1D-specific siRNAs (40 pmol), respectively. At 48 h post-transfection, 53BP1 (green) foci were immunostained using a 53BP1-specific antibody (catalog No. NB100-304, 1:1,000; Novus Biologicals). The nuclei were counterstained with DAPI (blue). The immunostaining was visualized, and images were captured using confocal microscopy. The red arrow-pointed immunostaining is representative of the prominent staining of 53BP1 throughout the entire nucleus. Scale bars = 20 μm. (D) The percentage of cells having ≥ five 53BP1 foci within an individual cell was calculated based on a total count of 300 cells. The data presented are the means of three independent experiments, with the error bars indicating the standard deviations of three independent experiments. Significance was determined using a Student’s *t*-test. EEF1D, eukaryotic translation elongation factor-1 δ; 53BP1, P53-binding protein 1; si-, small interfering; DAPI, 4′,6-diamidino-2-phenylindole. ^*^p < 0.05, ^***^p < 0.001.

The chromatin-associated protein 53BP1 is an important DDR signaling protein that plays a crucial role in the repair of DSBs [20,29]. Both 53BP1 protein expression and foci formation are altered during a BoAHV-1 infection *in vitro* and/or *in vivo* [18]. Nevertheless, it is unclear if EEF1D helps regulate 53BP1 signaling. Here, siRNA-mediated knockdown of EEF1D protein expression in MDBK cells increased 53BP1 protein expression (Fig. 4A). siEEF1D-1 and siEEF1D-2 increased the protein levels of 53BP1 by approximately 2.42- and 2.98-fold, respectively (Fig. 4B). Thus, EEF1D is potentially involved in regulating 53BP1 protein expression.

In response to DNA damage, 53BP1 rapidly forms nuclear foci, which are indispensable for exerting its DNA repair function [29,30]. The effects of the EEF1D protein on the formation of 53BP1 foci were examined. Mock-infected MDBK cells were transfected with EEF1D-specific siRNAs or scramble siRNAs and subjected to IFA assays. Consistent with the increased protein levels of 53BP1 after transfection with siEEF1D-1 and siEEF1D-2 (Fig. 4A and B), the intensity of 53BP1 nuclear foci significantly increased after EEF1D knockdown (Fig. 4C). Quantitative analysis suggested that approximately 21.20% of the control cells displayed ≥ 5 53BP1 foci. This proportion increased to 27.66% and 31.07% after transfection with siEEF1D-1 and siEEF1D-2, respectively (Fig. 4D). Prominent staining of 53BP1 throughout the entire nucleus was exclusively observed in a subset of MDBK cells transfected with either siRNA (Fig. 4C, indicated by red arrows). The intense immunostaining indicates interconnected 53BP1 foci. Thus, EEF1D knockdown enhanced 53BP1-mediated DDR signaling, providing evidence that EEF1D may regulate 53BP1 steady-state protein expression.

**Fig. 5:**
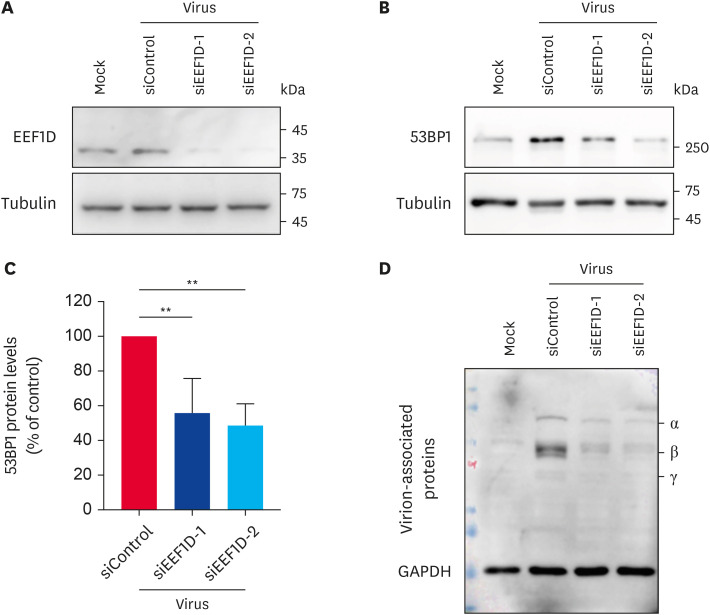
Effects of EEF1D on 53BP1 protein expression in virus-infected cells. MDBK cells in 6-well plates were transfected with scrambled siRNA, siEEF1D-1, and siEEF1D-2, at a dose of 150 pmol, respectively. At 36 h post-transfection, the cells were infected with BoAHV-1 at an MOI of 1. After infection for 24 h, the cells were collected for Western blot analysis using an antibody against EEF1D (catalog No. A2509, 1:1,000; Abclonal Technology) (A), 53BP1 P1 (catalog No. NB100-304, 1:3,000; Novus Biologicals) (B), and goat anti-BoAHV-1 serum (catalog No. PAB-IBR, 1:5,000; VMRD) (D), respectively. The data shown are representative of three independent experiments. (C) Band intensities were quantified using the free software Image J. The intensity of the 53BP1 band was first normalized to that of the respective loading control β-tubulin, and then normalized to that of the control transfected with scramble siRNA, which was arbitrarily set to 100%. The data presented are the means of three independent experiments, with error bars indicating the standard deviations of three independent experiments. Significance was determined using a Student’s *t*-test. EEF1D, eukaryotic translation elongation factor-1 δ; 53BP1, P53-binding protein 1; BoAHV-1, bovine alphaherpesvirus 1; MOI, multiplicity of infection; GAPDH, glyceraldehyde 3-phosphate dehydrogenase. ^**^p < 0.01.

These EEF1D-specific siRNAs significantly reduced EEF1D protein expression in the context of viral infection (Fig. 5A). Nevertheless, the effects of EEF1D knockdown had on 53BP1 protein expression observed in mock-infected MDBK cells (Fig. 4A), was reversed after a viral infection (Fig. 5B). Specifically, compared to cells transfected with scrambled siRNA, the 53BP1 protein levels decreased to approximately 55.87% and 48.82% in cells transfected with siEEF1D-1 and siEEF1D-2, respectively (Fig. 5C). Accordingly, siRNA-mediated depletion of EEF1D protein also leads to the reduction of viral replication as shown by the decreased protein expression levels of virion-associated proteins (Fig. 5D, denoted by α, β, and γ). Thus, the viral replication process may have greater capacity than EEF1D alone to influence 53BP1 protein expression.

These findings suggest that EEF1D depletion decreases 53BP1 steady-state expression in virus-infected cells. Foci formation was not evaluated under these conditions because BoAHV-1 productive infection at the late stages abolishes the formation of 53BP1 foci [18], and EEF1D knockdown leads to a decrease in 53BP1 protein levels (Fig. 5). Overall, the data show that EEF1D is potentially implicated in regulating 53BP1 signaling, a process that is disrupted by viral infection.

## DISCUSSION

Beyond its canonical role in facilitating protein synthesis [22], EEF1D influences diverse cellular processes, including the recognition of damaged proteins, activation of the proteasome degradation system, and control of apoptosis in physical conditions [12]. On the other hand, accumulating evidence also implicates EEF1D in the pathogenicity of various diseases, particularly in the progression, proliferation, and invasion of various cancers, such as gastric cancer and osteosarcoma [31,32]. A recent study showed that EEF1D promotes productive infection with BoAHV-1, most likely by facilitating formation of viral replication compartments through interaction with the viral protein ICP8, thereby implicating EEF1D in the viral replication process [14]. Here, EEF1D also functions as a potential modulator of DDR, in response to UV-irradiation-induced DNA damage (Fig. 1), and under conditions with or without a BoAHV-1 infection (Figs. 2 and 3). These findings align with a previous report that EEF1D depletion exacerbates DDP-induced DNA damage in ovarian cancer cells [19]. Thus, EEF1D is a versatile mediator of the DDR, active after UV irradiation and DDP treatment and during BoAHV-1 productive infection.

As a crucial molecule involved in the DDR, 53BP1 is indispensable for DSB repair [33]. Acute viral infection and reactivation from latency lead to the depletion of 53BP1 protein in the neurons of the trigeminal ganglia, a primary site for establishing lifelong latency, as characterized using a rabbit model [18]. Here, EEF1D depletion in uninfected cells increases the steady-state abundance of 53BP1, whereas this rise is abolished upon virus infection (Figs. 4 and 5). Hence, EEF1D normally restrains 53BP1 expression, but the virus infection overrides this control. Accordingly, reduced 53BP1 availability may compromise its potency in DSBs repair because γH2AX foci are further accumulated after EEF1D knockdown (Fig. 3), suggesting that DNA damage is intensified. Collectively, the data in the present study uncover a previously unrecognized role for EEF1D in governing 53BP1-dependent DDR, particularly in the context of BoAHV-1 infection. Elucidating the precise mechanisms through which EEF1D modulates 53BP1 remains an important avenue for future research.

A BoAHV-1 productive infection in a cell culture suppresses mitochondrial biogenesis [34,35], resulting in the excessive production of the inflammatory mediators reactive oxygen species that likely amplify viral pathogenicity [36]. A fraction of EEF1D was localized to the mitochondria [14], raising the intriguing question of whether EEF1D supports mitochondrial biogenesis by repairing mitochondrial DNA damage, a possibility warranting further investigation. As a potential oncolytic virus, BoAHV-1 can infect a broad range of human tumor cells while sparing healthy cells [37,38]. On the other hand, the mechanisms through which BoAHV-1 suppresses tumor cell proliferation and growth are unclear. A disruption of the host DNA-repair machinery partially accounts for the oncolytic effects of various oncolytic viruses [39]. This is similar to the mechanism of widely used chemotherapeutic drugs, such as olaparib and azacytidine [40,41]. These findings suggest that EEF1D may help regulate the host DDR, providing a novel perspective on the oncolytic effects of BoAHV-1, which warrants further study.

In summary, this paper provides the first evidence that EEF1D plays a crucial role in alleviating DNA damage induced by BoAHV-1 productive infection, potentially by regulating 53BP1-related DDR. These findings will extend the understanding of how viral infection perturbs host DDR signaling and position EEF1D as a novel host factor that affects viral pathogenesis.
